# Factors related to Japanese internal medicine doctors’ retention or migration to rural areas: a nationwide retrospective cohort study

**DOI:** 10.1265/ehpm.22-00169

**Published:** 2023-02-03

**Authors:** Yasuaki Saijo, Eiji Yoshioka, Yukihiro Sato, Yuki Kunori

**Affiliations:** Division of Public Health and Epidemiology, Department of Social Medicine, Asahikawa Medical University

**Keywords:** Internal medicine doctor, Rural medicine, Primary care, Board certification, Subspecialty

## Abstract

**Background:**

Internal medicine (IM) doctors in Japan play the role of primary care physicians; however, the shortage of rural physicians continues. This study aims to elucidate the association of age, sex, board certification, type of work, and main clinical work with the retention or migration of IM doctors to rural areas.

**Methods:**

This retrospective cohort study included 82,363 IM doctors in 2010, extracted from the national census data of medical doctors. The explanatory variables were age, sex, type of work, primary clinical work, and changes in board certification status. The outcome was retention or migration to rural areas. The first tertile of population density (PD) of municipalities defined as rural area. After stratifying the baseline ruralities as rural or non-rural areas, the odds ratios (ORs) of the explanatory variables were calculated using generalized estimation equations. The analyses were also performed after age stratification (<39, 40–59, ≥60 years old).

**Results:**

Among the rural areas, women had a significantly higher OR for retention, but obtaining board certification of IM subspecialties had a significantly lower OR. Among the non-rural areas, physicians who answered that their main work was IM without specific subspecialty and general had a significantly higher OR, but obtaining and maintaining board certification for IM subspecialties had a significantly lower OR for migration to rural areas. After age stratification, the higher OR of women for rural retention was significant only among those aged 40–59 years. Those aged under 40 and 40–59 years in the non-rural areas, who answered that their main work was IM without specific subspecialty had a significantly higher OR for migration to rural areas, and those aged 40–59 years in the rural areas who answered the same had a higher OR for rural retention.

**Conclusions:**

Obtaining and maintaining board certification of IM subspecialties are possible inhibiting factors for rural work, and IM doctors whose main work involves subspecialties tend to work in non-rural areas. Once rural work begins, more middle-aged female IM doctors continued rural work compared to male doctors.

**Supplementary information:**

The online version contains supplementary material available at https://doi.org/10.1265/ehpm.22-00169.

## Introduction

Primary care doctors improve the outcomes of chronic disease care, decrease hospitalization and the use of emergency department visits, and play a gatekeeping role [1–3]. As Japan has not yet adopted a general practitioner system, patients can see any physician free of cost, and licensed medical doctors with any specialty can open clinics anywhere [4]. In Japan, where no further certificate besides a medical license is needed to become a primary care doctor, internal medicine (IM) doctors play an important role in primary care in Japan [5]. However, the shortage of primary care doctors in rural areas has become a problem [6], similar to the worldwide shortage of healthcare workers in rural areas [7].

Many Japanese physicians have attempted to obtain board certification, although this does not increase medical fees in Japan’s health insurance system. Newly board-certified physicians in rural and intermediate municipalities are likely to migrate to urban municipalities [8]; however, the participants in this study were all physicians, including those with specialties unsuitable for rural areas, and the follow-up period was only two years. To increase the number of rural physicians, the regional quota program at medical schools began in 2008, and medical students who take exams through the regional quota program and receive student loans, are obliged to work for the prefecture for nine years instead of exempting repayment [9]. A follow-up study reported that medical students with scholarships from the regional quota program worked more significantly in non-metropolitan areas for five years after graduation [10]. Although there is limited evidence on the short-term effect, the long-term effect, especially after the obligatory term, remains unknown. While primary care has mainly been provided by IM doctors in Japan as family medicine doctors are not popular, the Japan Primary Care Association introduced board certification for primary care physicians in 2009 to improve the quality of primary care. Family physicians certified by the Japan Primary Care Association worked in more rural areas than other physicians [11], but their numbers were small (N = 900 on Sept 30, 2020) [12]. Furthermore, the Japan Medical Association has proposed *Kakaritsuke* physicians, defined as “a locally-based and reliable physician with comprehensive capabilities in community health, public health and welfare, who is available for consultation about any health issues, has a good understanding of advanced healthcare information, and can refer patients to specialists or specialized healthcare facilities when needed” [13]. Several IM doctors have been assigned to *Kakaritsuke* physicians for regional medicine.

Thus, IM doctors in Japan play a major role in primary care; however, the shortage of rural physicians continues, and the factors affecting rural retention and migration among IM doctors should be elucidated. This study aimed to elucidate the association of age, sex, board certification, type of work, and main clinical work of IM doctors with their retention or migration to rural areas using longitudinal data from the Survey of Physicians, Dentists, and Pharmacists, a national biennial census survey of medical doctors.

## Methods

A retrospective cohort study design was applied, and data from the Survey of Physicians, Dentists, and Pharmacists (2010, 2012, 2014, and 2016), a biennial national census conducted by the Japanese Ministry of Health, Labour, and Welfare (MHLW) was used. All licensed physicians, dentists, and pharmacists in Japan are obligated to register for the survey based on the Medical Practitioners’ Act. Permission to use the datasets from the MHLW was obtained (Statistics Law, Article 33), and this study was approved by the Asahikawa Medical University Research Ethics Committee (approval number: 21104 (Oct 13, 2021)).

**Fig. 1:**
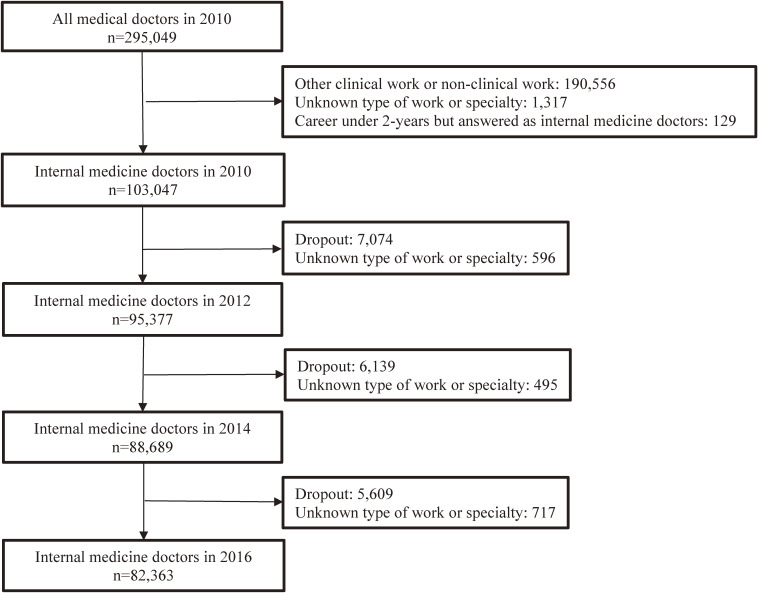
Flow chart of the study participants

The baseline cohort was comprised of IM doctors working in clinics, hospitals, or medical schools in 2010. IM doctors were defined as those who reported that their main specialties were IM (no specific subspecialties selected), general, or an IM subspecialty, including respiratory medicine, cardiovascular medicine, gastroenterology, renal medicine, neurology, diabetology, and infectious diseases. Physicians with only two years of experience were excluded because they were junior residents and were asked to indicate their specialty as a “junior resident.” Of the 295,049 registered doctors, the number of IM doctors in 2010 was 103,047. After excluding those who dropped out or did not respond to the type of work or specialty in 2012, 2014, or 2016, the final sample size was 82,363 (Fig. 1).

The outcome was retention or migration to rural areas. The data included the municipality of the workplace, and the population density (PD) of each municipality was calculated based on the 2015 census data. The first, second, and third tertiles of population density were categorized as rural, intermediate, and urban areas, respectively [8]. The number of municipalities was 1,741, and the cutoff points were 88.0 and 447.3/km^2^, respectively.

The explanatory variables were age, sex, type of work, primary clinical work, and changes in board certification status. Age was categorized as ≤29, 30–34, 35–39, 40–44, 45–49, 50–54, 55–59, 60–64, and ≥65 years old. The main clinical work in 2010 (baseline) was categorized as IM, IM subspecialty, and general. The type of work was categorized as clinic founder or director, hospital founder or director, clinic staff, hospital staff, clinical faculty member of medical school, and clinical staff or clinical medicine PhD student of medical school.

Changes from the previous two years in the certification status of the Fellowship of the Japanese Society of IM were categorized as not certified continuously, maintained, newly certified, and dropped out in 2012, 2014, and 2016. Since its inception, board certification in Japan is operated by academic societies; however, the new board certification system training started in 2018 to coordinate and harmonize the criteria. The process is led by the Panel on Board Certification within the MHLW, and, in addition to IM, the following have been defined as general areas of board certification in Japan: surgery, pediatrics, obstetrics and gynecology, orthopedics, neurosurgery, ophthalmology, otorhinolaryngology, acute medicine, anesthesiology, dermatology, urology, plastic surgery, radiology, pathology, rehabilitation, psychiatry, laboratory medicine, and general practice [8]. Because board certification before the new system began remain valid after the new system started, the general area definition was used for general area board certifications other than general IM (double or more certifications in the general area were allowed in the old system and partially so in the new system). However, because laboratory medicine and general practice were not asked, and psychiatry was asked only in the 2014 and 2016 surveys, they were not used. Thus, the changes in the number of board-certified general areas other than general IM two years prior were categorized as not certified continuously, maintained, obtaining, and losing board certification in 2012, 2014, and 2016. The IM subspecialties included are as follows: gastroenterology, cardiology, respirology, hematology, endocrinology, diabetology, nephrology, hepatology, allergology, infectious diseases, gerontology, neurology, rheumatology, gastroenterological endoscopy, and medical oncology, as defined by the Japanese Medical Specialty Board [14].

### Statistical analysis

For the longitudinal data with three repeated measures of outcome and time-varying variables, the random-effects logistic regression generalized estimating equation (GEE) was used. The GEE considers repeated measures, to estimate the adjusted odds ratios (ORs) of retention or migration to rural areas after stratifying the baseline PD ruralities as rural and non-rural area (intermediate and urban area). The outcomes were retention and migration to rural areas in 2012, 2014, and 2016, respectively. The explanatory variables included at the baseline (2010) were age, sex, type of work, and main clinical work. The explanatory variables included during the follow-up periods (2012, 2014, and 2016) were changes in board certification status of general IM, IM, changes in the number of board certifications of IM subspecialties, and changes in the number of general area board certifications other than general IM. Analyses were also performed after age stratification (≤39, 40–59, ≥60 years). Statistical significance was set at P < 0.05. All analyses were conducted using the Stata statistical software version 17.0 for Windows (StataCorp, College Station, TX, USA).

## Results

Table 1 lists the number of IM doctors per year. The number of IM doctors in the first tertile of PD areas declined from 3,744 to 3,290 over the six years. In the baseline survey (2010), most participants were men (85.4%), the largest main clinical work was IM (57.5%, subspecialties or general not selected), and the largest type of work was hospital staff (42.5%). The percentage of board certifications for general IM was 14.3%, with 38.8% having one or more IM subspecialty board certification, and 5.0% with one or more general area certification other than general IM (Table 2). The movements between municipalities are shown in Figure S1, and the follow-up results for board certification are shown in Table S1.

**Table 1:** Number of internal medicine doctors in 2010, 2012, 2014 and 2016 (N = 82,363)

	**2010**		**2012**		**2014**		**2016**	
	**N**	**%**	**N**	**%**	**N**	**%**	**N**	**%**
Population density								
1st tertile	3,744	4.6	3,515	4.3	3,412	4.1	3,290	4.0
2nd tertile	16,030	19.5	15,438	18.7	15,213	18.5	15,080	18.3
3rd tertile	62,589	76.0	59,727	72.5	58,981	71.6	58,460	71.0
Not working as IM doctors	—	—	3,683	4.5	4,757	5.8	5,533	6.7

IM: internal medicine doctor

**Table 2:** Characteristics of Internal medicine doctors (Year 2010)

	**All** **(N = 82,363)**		**<40 years** **(N = 21,035)**		**40–59 years** **(N = 43,793)**		**≥65 years** **(N = 17,535)**	
			
	**N**	**%**	**N**	**%**	**N**	**%**	**N**	**%**
Sex								
Men	70,347	85.4	15,882	75.5	38,140	87.1	16,325	93.1
Women	12,016	14.6	5,153	24.5	5,653	12.9	1,210	6.9
Age								
–29	3,837	4.7	3,837	18.2	–	–	–	–
30–34	8,060	9.8	8,060	38.3	–	–	–	–
35–39	9,138	11.1	9,138	43.4	–	–	–	–
40–44	10,256	12.5	–	–	10,256	23.4	–	–
45–49	11,609	14.1	–	–	11,609	26.5	–	–
50–54	11,935	14.5	–	–	11,935	27.3	–	–
55–59	9,993	12.1	–	–	9,993	22.8	–	–
60–64	7,643	9.3	–	–	–	–	7,643	9.3
65–	9,892	12.0	–	–	–	–	9,892	12.0
Main clinical work								
Internal medicine	47,331	57.5	6,438	30.6	26,526	60.6	14,367	81.9
Internal medicine sub-specialty	34,856	42.3	14,525	69.1	17,197	39.3	3,134	17.9
General	176	0.2	72	0.3	70	0.2	34	0.2
Type of work								
Clinic founder or director	25,174	30.6	528	2.5	14,515	33.1	10,131	57.8
Hospital founder or director	1,631	2.0	38	0.2	661	1.5	932	5.3
Clinic staff	9,550	11.6	1,478	7.0	5,841	13.3	2,231	12.7
Hospital staff	35,014	42.5	12,431	59.1	18,629	42.5	3,954	22.6
Clinical faculty member of medical school	6,401	7.8	2,318	11.0	3,809	8.7	274	1.6
Clinical staff or PhD student of Medical School	4,593	5.6	4,242	20.2	338	0.8	13	0.1
Board certification of general internal medicine	11,773	14.3	1,515	7.2	8,877	20.3	1,381	7.9
Number of board certifications of internal medicine subspeciality								
0	50,362	61.2	15,132	71.9	22,414	51.2	12,816	73.1
1	21,698	26.3	4,282	20.4	14,158	32.3	3,258	18.6
2	8,191	9.9	1,323	6.3	5,666	12.9	1,202	6.9
3	1,947	2.4	284	1.4	1,438	3.3	225	1.3
4	124	0.2	11	0.1	92	0.2	21	0.1
5	28	0.0	2	0.0	17	0.0	9	0.1
6	7	0.0	–	–	3	0.0	4	0.0
7	4	0.0	1	0.0	3	0.0	–	–
8	1	0.0	–	–	1	0.0	–	–
9	1	0.0	–	–	1	0.0	–	–
Number of general area board certifications other than general internal medicine								
0	78,265	95.0	20,737	98.58	41,103	93.9	16,425	93.7
1	3,940	4.8	294	1.4	2,607	6.0	1,039	5.9
2	146	0.2	4	0.02	78	0.2	64	0.4
3	10	0.0	–	–	4	0.0	6	0.0
4	1	0.0	–	–	1	0.0	–	–
5	1	0.0	–	–	–	–	1	0.0

Table 3 shows the ORs for retention or migration to most rural areas stratified by the PD rurality definition as rural and non-rural areas. Among the rural areas, women and older age groups had a significantly higher OR. Clinic founders or directors and hospital founders or directors had a significantly higher OR, but hospital staff had a significantly lower OR. Regarding changes in board certification status, a new certification of general IM and obtaining certifications of IM subspecialties had a significantly lower OR, and maintaining, obtaining, and losing certifications of general area other than general IM had a significantly lower OR. Among the non-rural areas, women and older age groups had a significantly lower OR. Physicians who answered that their main work was IM and general had a significantly higher OR. Clinic founders or directors and hospital founders or directors had a significantly lower OR, but hospital staff, clinical faculty member of medical school, and clinical staff or PhD students in medical schools had a significantly higher OR. As for the change in board certification status, maintaining, obtaining, and losing certifications of IM subspecialties had a significantly lower OR.

**Table 3:** ORs for working at first tertile PD municipalities (rural area)

	**1st tertile population density municipalities (rural area)**					**2nd and 3rd tertile population density municipalities (non-rural area)**				
	
	**OR**	**95% CI**			**P**	**OR**	**95% CI**			**P**
Sex										
Male	1.00					1.00				
Female	1.77	1.03	—	3.04	0.039	0.30	0.21	—	0.44	<0.001
Age (years)										
≤29	0.05	0.02	—	0.12	<0.001	2.28	1.46	—	3.58	<0.001
30–34	0.04	0.02	—	0.08	<0.001	1.69	1.15	—	2.47	0.007
35–39	1.00					1.00				
40–44	5.48	2.70	—	11.09	<0.001	0.57	0.37	—	0.88	0.012
45–49	14.07	6.93	—	28.56	<0.001	0.66	0.43	—	1.01	0.053
50–54	17.23	8.66	—	34.29	<0.001	0.42	0.26	—	0.68	<0.001
55–59	14.71	7.17	—	30.19	<0.001	0.38	0.22	—	0.66	0.001
60–64	12.72	6.01	—	26.93	<0.001	0.53	0.30	—	0.94	0.031
≥65	4.92	2.41	—	10.05	<0.001	0.24	0.13	—	0.46	<0.001
Primary clinical work										
Internal medicine	1.56	0.99	—	2.46	0.057	1.54	1.17	—	2.01	0.002
Subspecialty	1.00					1.00				
General	1.81	0.37	—	8.75	0.462	10.71	1.61	—	71.12	0.014
Type of work										
Clinic founder or director	20.91	11.47	—	38.10	<0.001	0.02	0.01	—	0.03	<0.001
Clinic staff	1.00					1.00				
Hospital founder or director	3.30	1.27	—	8.58	0.014	0.08	0.01	—	0.46	0.005
Hospital staff	0.57	0.37	—	0.87	0.009	2.30	1.47	—	3.59	<0.001
Clinical faculty member of medical school	0.11	0.01	—	1.23	0.073	1.87	1.03	—	3.37	0.038
Clinical staff or PhD student of Medical School	0.15	0.01	—	2.78	0.202	4.24	2.39	—	7.50	<0.001
Changes in board certification status of general internal medicine										
Not certified continuously	1.00					1.00				
Maintained	0.66	0.38	—	1.14	0.136	0.72	0.49	—	1.06	0.092
Newly certified	0.37	0.23	—	0.59	<0.001	0.79	0.55	—	1.15	0.226
Dropped out	0.64	0.32	—	1.29	0.213	1.00	0.53	—	1.87	0.993
Changes in the number of board certifications of internal medicine subspeciality										
No certified continuously	1.00					1.00				
Maintained	0.93	0.63	—	1.39	0.734	0.49	0.37	—	0.63	<0.001
Obtained	0.58	0.38	—	0.90	0.016	0.36	0.27	—	0.49	<0.001
Lost	0.61	0.35	—	1.08	0.090	0.62	0.40	—	0.95	0.026
Changes in the number of general area board certifications other than general internal medicine										
No certified continuously	1.00					1.00				
Maintained	0.16	0.09	—	0.30	<0.001	0.71	0.36	—	1.40	0.318
Obtained	0.07	0.03	—	0.15	<0.001	0.86	0.36	—	2.04	0.730
Lost	0.23	0.11	—	0.50	<0.001	1.04	0.41	—	2.63	0.933

OR: odds ratio, CI: confidence interval

Table 4-1 shows the ORs among IM doctors aged <40 years. Among the rural areas, clinic founders or directors had a significantly higher OR. Regarding changes in board certification status, a new certification of general IM, and maintaining and obtaining certifications of IM subspecialties, and maintaining, and obtaining certifications of general area other than general IM had a significantly lower OR. Among the non-rural areas, women had a significantly lower OR. Physicians who answered that their main work was IM and general had a significantly higher OR. Clinic/hospital founders or directors had a significantly lower OR, but hospital staff, and clinical staff or PhD students in medical schools had a significantly higher OR. As for the change in board certification status, maintaining certification of general IM, and maintaining, obtaining, and losing certifications of IM subspecialties had a significantly lower OR.

**Table 4-1:** ORs for working at rural area among IM doctors aged under 40 years

	**1st tertile population density municipalities (rural area)**					**2nd and 3rd tertile population density municipalities (non-rural area)**				
	
	**OR**	**95%CI**			**P**	**OR**	**95%CI**			**P**
Sex										
Male	1.00					1.00				
Female	1.07	0.53	—	2.14	0.858	0.39	0.26	—	0.58	<0.001
Age (years)										
<29	0.06	0.03	—	0.15	<0.001	2.01	1.30	—	3.10	0.002
30–34	0.06	0.03	—	0.12	<0.001	1.68	1.16	—	2.43	0.006
35–39	1.00					1.00				
Primary clinical work										
Internal medicine	0.98	0.50	—	1.95	0.964	1.89	1.32	—	2.69	<0.001
Subspecialty	1.00					1.00				
General	2.01	0.34	—	11.95	0.444	17.75	1.53	—	206.02	0.021
Type of work										
Clinic founder or director*	28.59	3.31	—	246.93	0.002	0.02	0.00	—	0.43	0.011
Clinic staff	1.00					1.00				
Hospital founder or director	10.75	0.13	—	907.41	0.294	—**				
Hospital staff	0.80	0.39	—	1.65	0.552	2.71	1.19	—	6.17	0.018
Clinical faculty member of medical school	—					2.24	0.85		5.92	0.103
Clinical staff or PhD student of Medical School	0.18	0.01	—	2.50	0.199	5.06	2.10	—	12.21	<0.001
Changes in board certification status of general internal medicine										
No certified continuously	1.00					1.00				
Maintain	0.49	0.19	—	1.27	0.140	0.42	0.21	—	0.85	0.016
Newly certified	0.50	0.27	—	0.93	0.029	0.67	0.42	—	1.06	0.086
Dropped out	0.63	0.16	—	2.45	0.509	0.88	0.29	—	2.68	0.828
Changes in the number of board certifications of internal medicine subspeciality										
No certified continuously	1.00					1.00				
Maintained	0.45	0.23	—	0.90	0.023	0.48	0.34	—	0.68	<0.001
Obtained	0.35	0.19	—	0.65	0.001	0.32	0.23	—	0.45	<0.001
Lost	0.73	0.24	—	2.20	0.573	0.39	0.19	—	0.81	0.011
Changes in the number of general area board certifications other than general internal medicine										
No certified continuously	1.00					1.00				
Maintained	0.08	0.02	—	0.33	0.001	1.33	0.43	—	4.06	0.621
Obtained	0.06	0.01	—	0.30	0.001	1.48	0.44	—	5.04	0.528
Lost^#^	4.28	0.14	—	127.28	0.401	—**				

OR: odds ratio, CI: confidence interval

*In non-rural area analysis, this category was merged with “hospital founder or director”

#In non-rural area analysis, this category was merged with “No certified continuously”

**No participants migrated to rural area

Table 4-2 shows the ORs among IM doctors aged 40–59 years. Among the rural areas, women had a significantly higher OR. A significantly higher OR of physicians who answered that their main work was IM was found. Clinic founders or directors had a significantly higher OR, but hospital staff had a significantly lower OR. Regarding changes in board certification status, a new certification of general IM, and maintaining, obtaining, and losing certifications of general area other than general IM had a significantly lower OR. Among the non-rural area, women had a significantly lower OR. Physicians who answered that their main work was IM had a significantly higher OR. Clinic founders or directors had a significantly lower OR, but hospital staff, and clinical staff or PhD students of a medical school had a significantly higher OR. As for the change in board certification status, maintaining and obtaining certifications of IM subspecialties had a significantly lower OR.

**Table 4-2:** ORs for working at rural area among IM doctors aged from 40 to 59 years

	**1st tertile population density municipalities (rural area)**					**2nd and 3rd tertile population density municipalities (non-rural area)**				
	
	**OR**	**95%CI**			**P**	**OR**	**95%CI**			**P**
Sex										
Male	1.00					1.00				
Female	4.04	1.65	—	9.90	0.002	0.18	0.07	—	0.46	<0.001
Age (years)										
40–44	1.00					1.00				
45–49	2.97	1.41	—	6.26	0.004	1.14	0.73	—	1.79	0.555
50–54	3.54	1.72	—	7.25	0.001	0.74	0.45	—	1.22	0.238
55–59	3.06	1.43	—	6.55	0.004	0.69	0.39	—	1.20	0.192
Primary clinical work										
Internal medicine	2.26	1.15	—	4.43	0.018	1.51	1.01	—	2.26	0.046
Subspecialty	1.00					1.00				
General	0.91	0.04	—	22.50	0.952	2.61	0.09	—	78.47	0.581
Type of work										
Clinic founder or director	34.81	13.46	—	90.05	<0.001	0.02	0.01	—	0.04	<0.001
Clinic staff	1.00					1.00				
Hospital founder or director	3.05	0.64	—	14.50	0.161	0.08	0.00	—	2.24	0.135
Hospital staff	0.52	0.27	—	0.98	0.043	2.38	1.35	—	4.20	0.003
Clinical faculty member of medical school	0.07	0.00	—	1.66	0.101	1.78	0.80	—	3.94	0.158
Clinical staff or PhD student of Medical School	—					6.34	1.55	—	25.91	0.010
Changes in board certification status of general internal medicine										
No certified continuously	1.00					1.00				
Maintain	0.91	0.42	—	1.98	0.818	0.98	0.61	—	1.58	0.942
Newly certified	0.32	0.15	—	0.70	0.005	0.92	0.48	—	1.74	0.788
Dropped out	0.68	0.25	—	1.88	0.456	1.17	0.52	—	2.66	0.701
Changes in the number of board certifications of internal medicine subspeciality										
No certified continuously	1.00					1.00				
Maintained	1.14	0.63	—	2.07	0.669	0.47	0.31	—	0.72	<0.001
Obtained	0.80	0.38	—	1.71	0.566	0.51	0.29	—	0.91	0.023
Lost	0.74	0.32	—	1.74	0.495	0.70	0.38	—	1.29	0.251
Changes in the number of general area board certifications other than general internal medicine										
No certified continuously	1.00					1.00				
Maintained	0.12	0.05	—	0.32	<0.001	0.54	0.21	—	1.40	0.207
Obtained	0.07	0.02	—	0.26	<0.001	0.90	0.24	—	3.33	0.879
Lost	0.21	0.06	—	0.67	0.009	1.82	0.64	—	5.16	0.260

OR: odds ratio, CI: confidence interval

Table 4-3 shows the ORs among IM doctors aged 60 or more years. Among the rural areas, clinic founders or directors had a significantly higher OR. Regarding changes in board certification status, a new certification of general IM, and obtaining, and losing certifications of general area other than general IM had a significantly lower OR. Among the non-rural areas, clinic, women had a significantly lower OR. Clinic founders or directors and hospital founders or directors had a significantly lower OR.

**Table 4-3:** ORs for working at rural area among IM doctors aged 60 or more years

	**1st tertile population density municipalities (rural area)**						**2nd and 3rd tertile population density municipalities (non-rural area)**				
	
	**OR**	**95%CI**				**P**	**OR**	**95%CI**			**P**
Sex											
Male	1.00						1.00				
Female	1.07	0.25	—	4.50		0.930	0.02	0.00	—	0.27	0.004
Age (years)											
60–64	1.00						1.00				
≥65	0.45	0.24	—	0.85		0.014	0.52	0.24	—	1.13	0.097
Primary clinical work											
Internal medicine	1.52	0.40	—	5.75		0.534	0.76	0.30	—	1.94	0.565
Subspecialty	1.00						1.00				
General	0.31	0.01	—	17.88		0.575	7.73	0.07	—	843.58	0.393
Type of work											
Clinic founder or director*	10.43	4.43	—	24.58		<0.001	0.01	0.00	—	0.02	<0.001
Clinic staff	1.00						1.00				
Hospital founder or director	2.15	0.59	—	7.85		0.247	0.04	0.00	—	0.47	0.011
Hospital staff	0.46	0.19	—	1.11		0.086	1.49	0.55	—	4.06	0.430
Clinical faculty member of medical school	0.08	0.00	—	29.95		0.398	1.69	0.23	—	12.45	0.609
Clinical staff or PhD student of Medical School	—						—**				
Changes in board certification status of general internal medicine											
No certified continuously	1.00						1.00				
Maintain	0.26	0.05	—	1.41		0.118	0.63	0.09	—	4.20	0.629
Newly certified	0.17	0.04	—	0.68		0.012	1.84	0.35	—	9.69	0.470
Dropped out	0.45	0.09	—	2.21		0.328	0.87	0.14	—	5.52	0.881
Changes in the number of board certifications of internal medicine subspeciality											
No certified continuously	1.00						1.00				
Maintained	2.42	0.93	—		6.35	0.072	0.53	0.20	—	1.39	0.197
Obtained	1.12	0.33	—		3.89	0.853	0.39	0.09	—	1.77	0.223
Lost	0.48	0.16	—		1.40	0.179	1.22	0.41	—	3.66	0.722
Changes in the number of general area board certifications other than general internal medicine											
No certified continuously	1.00						1.00				
Maintained	0.42	0.14	—		1.30	0.132	0.93	0.14	—	6.24	0.942
Obtained	0.08	0.03	—		0.25	<0.001	0.13	0.01	—	2.48	0.173
Lost	0.24	0.08	—		0.71	0.010	0.32	0.02	—	6.39	0.454

OR: odds ratio, CI: confidence interval

*In non-rural area analysis, this category was merged with “Clinical staff or PhD student of Medical School”

**No participants migrated to rural area

## Discussion

In this retrospective cohort study using national survey data on Japanese IM doctors, we found that general IM work and the two-year changes in board certification status was a significant factor in their retention and migration to rural areas. To the best of our knowledge, this is the first nationwide longitudinal study to report the factors for retention and migration to rural areas among Japanese IM doctors, who play an important role in rural primary care.

Physicians who mainly work for IM subspecialties are generally distributed in urban areas more than general IM doctors are [15]. Maintaining and obtaining board certification of IM subspecialties was negatively associated with rural migration among the rural area municipalities. Furthermore, among the rural area municipalities, new board certifications of IM subspecialties were negatively associated with retention. This result may reflect migration to urban areas where specialties can enable physicians to perform well. In the age-stratified analysis, the significance of changes in certification of IM subspecialty among IM doctors aged ≥60 in the rural area municipalities disappeared. This may indicate that the importance of IM subspecialty board certifications is lower for older doctors. Moreover, among the non-rural area municipalities, physicians answered that their main work was IM without specific specialty and general, had a significantly higher OR for migration to rural areas, and in age-stratified analysis, those aged <40 years had the same result, and those aged 40–59 years who answered IM with specific specialty had a higher OR for rural retention. Thus, IM doctors who work with general internal medicine may have more rural incentives.

Concerning the changes in board certification status of general IM, new certification was negatively associated with retention in rural areas among the rural area municipalities, and the associations were consistent even after age stratification. The new certification allowing rural physicians to migrate to non-rural areas may mean that they want to gain or maintain the board certification of IM subspecialties for which training is more accessible in urban areas. Therefore, because certified family physicians (started in 2009) are considered to correspond to physicians who answered “general,” their increasing numbers are expected to be helpful for rural medicine [11]. Meanwhile, IM subspecialties may be linked to working in non-rural areas. Since the start of the new board certification system training in 2018, there has been a concern in Japan that younger physicians seeking specialist careers lead to fewer physicians in rural areas [8].

Maintaining and obtaining among the rural area municipalities is associated with lower retention, and the age-stratified results were almost consistent with the overall IM doctors analysis. Since some general areas, such as radiology, anesthesiology, emergency medicine, and pathology, were originally common in urban areas [15], IM doctors who had other general area board certifications and intended to utilize them seemingly needed to move to non-rural areas. Moreover, losing general area board certifications, other than general IM, in the rural area municipalities was significantly associated with lower retention in rural areas. While the reasons are unknown, it may be because the follow-up study for the previous two years among all physicians reported that the loss of general area board certification among rural physicians was significantly related to urban migration, and the timing of the change in board certification status is a possible time to intervene and improve physician distribution [8]. However, the new board certification system in Japan restricts double general boards for general IM, except for family physicians [16] because double boards in a general area cannot guarantee quality [17]. Thus, the influence of double general area boards on the distribution of rural IM doctors seems to have declined.

In the non-rural area municipalities, female physicians had lower ORs for migration to rural areas, and the age-stratified ORs were consistent with the results for IM doctors overall. However, those in the rural areas had a higher OR for retention in rural areas, and the age-stratified results revealed that higher rural retention was only significant among those aged 40–59 years. It has been reported that more female physicians work in urban areas than male physicians [18, 19], and rural sociocultural factors may preclude women from accepting decision-making positions [20]. However, once female physicians start working in rural areas, they tend to continue doing so, unlike male physicians; however, those who are able to start rural work may have stronger intentions to commit to rural medicine.

In this study, the retention of younger physicians in rural areas was low and migration to non-rural areas was high. These results are compatible with reports of higher migration rates among younger physicians [21, 22].

IM doctors at medical schools in non-rural areas had a higher OR for migration to rural areas. In Japan, many medical graduates have joined a clinical specialty department (known as *Ikyoku*) at their university to secure employment for new graduates, and *Ikyoku* generally dispatches physicians to affiliated hospitals, including rural ones. Thus, *Ikyoku* plays an important role in deploying physicians in rural areas [23]. The higher OR of non-rural hospital staff may be due to some of them being forcibly deployed in rural areas through *Ikyoku*. In contrast, clinic and hospital founders and directors were associated with lower migration to rural areas and higher retention in rural areas. In the Japanese health insurance system, opening self-employed clinics has high start-up costs for physicians [15], and it is difficult for clinic founders to move away from their clinics, reflecting a higher OR for retention in rural areas and a lower OR for migration to them. In the age-stratified analysis some ORs were not calculable because the number of clinical faculty members of medical schools among younger physicians and clinical staff or PhD students of medical schools among older physicians were small.

This study had several limitations. First, using fixed survey data, family [24], rural background [25], and regional quota medical students with scholarships [10] could have affected working in rural areas, and these factors were not considered in our study. We were unable to account for these factors in our analysis. Second, under the new board certification system, training that began in 2018, double boards of general areas, and the number of obtainable IM subspecialties were restricted. Therefore, the number of IM doctors with double boards or many subspecialty boards is declining. Third, because there has been no established rural definition for rural medicine [26], we used the rural definitions from the latest Japanese study on the rural work of physicians [8]. Fourth, the number of IM doctors during the follow-up period may have been underestimated because the dropout IM doctors may have returned to IM work later. However, we believe that the ORs were accurately estimated as the follow-up rate was 80%.

## Conclusion

Maintaining and obtaining board certifications of IM subspecialties are possible inhibiting factors for rural work, and IM doctors whose main work involves subspecialties tend to work in non-rural areas. Having general area board certifications other than general IM among rural IM doctors may be an inclination to migrate to non-rural areas. Therefore, family medicine education for medical students and residents should be increased to raise awareness of the importance of family medicine doctors who can do IM work in both rural and non-rural areas. Once rural work began, more middle-aged female IM doctors continued rural work compared to male doctors. IM doctors at medical schools have a high possibility of migration and are probable supply sources for rural physicians.

## Abbreviations

IM: internal medicine
OR: odds ratio
PD: population density
MHLW: Ministry of Health, Labor and Welfare
CI: confidence interval

## Supplementary information

**Additional file 1:** **Supplemental Table 1** Board certification changes in 2012, 2014, and 2016. **Figure S1** An overview of movements between municipalities among internal medicine physicians in Japan.

## Declarations

### Availability of data and material

The raw data collected for government statistics will not be shared because of the restrictions stipulated by the MHLW.
