# Supplementary material for: Factors related to Japanese internal medicine doctors’ retention or migration to rural areas: a nationwide retrospective cohort study
Source: Environ Health Prev Med. 2023 Feb 3;28:14. doi: 10.1265/ehpm.22-00169 (PMC9922564; doi:10.1265/ehpm.22-00169)
Supplement: Supplementary file 1 — Additional file 1: Supplemental Table 1 Board certification changes in 2012, 2014, and 2016. Figure S1 An overview of movements between municipalities among internal medicine physicians in Japan. [file ehpm-28-014-s001.docx]

Supplemental Table 1 Board certification changes in 2012, 2014, and 2016

|  | 2012 | |  | 2014 | |  | 2016 | |
| --- | --- | --- | --- | --- | --- | --- | --- | --- |
|  | N | % |  | N | % |  | N | % |
| Change of board certification of general internal medicine |  |  |  |  |  |  |  |  |
| Not certified continuously | 68,293 | 82.9 |  | 67,776 | 82.3 |  | 62,594 | 76.0 |
| Maintained | 9,373 | 11.4 |  | 9,977 | 12.1 |  | 11,706 | 14.2 |
| Newly certified | 2,297 | 2.8 |  | 2,917 | 3.5 |  | 6,875 | 8.4 |
| Dropped out | 2,400 | 2.9 |  | 1,693 | 2.1 |  | 1,188 | 1.4 |
| Change of number of board certifications of internal medicine subspeciality |  |  |  |  |  |  |  |  |
| Not certified continuously | 44,578 | 54.1 |  | 41,416 | 50.3 |  | 38,953 | 47.3 |
| Maintained | 26,541 | 32.2 |  | 29,483 | 35.8 |  | 33,027 | 40.1 |
| Obtained | 7,513 | 9.1 |  | 7,722 | 9.4 |  | 7,210 | 8.8 |
| Lost | 3,731 | 4.5 |  | 3,742 | 4.5 |  | 3,173 | 3.9 |
| Change of the number of general area board certifications other than general internal medicine |  |  |  |  |  |  |  |  |
| Not certified continuously | 77,494 | 94.1 |  | 77,814 | 94.5 |  | 77,935 | 94.6 |
| Maintained | 3,091 | 3.8 |  | 2,935 | 3.6 |  | 2,795 | 3.4 |
| Obtained | 790 | 1.0 |  | 653 | 0.8 |  | 830 | 1.0 |
| Lost | 988 | 1.2 |  | 961 | 1.2 |  | 803 | 1.0 |

2016

2014

2012

2010

1st tertile PDM

N=3, 774

2nd tertile PDM

N=16,030

3rd tertile PDM

N=16,030

1st tertile PDM

N=3,515

2nd tertile PDM

N=15,438

3rd tertile PDM

N=59,727

Other

N=3,683

1st tertile PDM

N=3,412

2nd tertile PDM

N=15,213

3rd tertile PDM

N=59,981

Other

N=4,753

1st tertile PDM

N=3,290

2nd tertile PDM

N=15,080

3rd tertile PDM

N=58,460

Other

N=5,533

a

b

c

d

e

f

a

b

c

d

e

f

a

b

c

d

e

f

g

g

|  | 2010-2012 | 2012-2014 | 2014-2016 |
| --- | --- | --- | --- |
| a | 3,103 | 3,032 | 2,987 |
| b | 193 | 125 | 108 |
| c | 238 | 231 | 204 |
| d | 210 | 127 | 113 |
| e | 164 | 133 | 101 |
| f | 248 | 196 | 152 |
| g | - | 51 | 50 |

Figure S1 An overview of movements between municipalities among internal medicine physicians in Japan.

The figure shows only movements from 1st tertile population density municipalities to others and from others to 1st tertile population density municipalities.

PDM: population density municipalities
